# Aberrant CD19 Expression on CD8‐Positive T‐Cell Lymphoma

**DOI:** 10.1111/ijlh.70098

**Published:** 2026-03-25

**Authors:** Brian Vadasz, Yijie Liu, Qing Chen

**Affiliations:** ^1^ Department of Pathology, Northwestern Memorial Hospital Northwestern University Feinberg School of Medicine Chicago Illinois USA

Fifty‐nine‐year‐old male with a history of primary CD30+ cutaneous T‐cell lymphoma (CTCL) presented with difficulty in eating and a mass at the base of the tongue. Biopsy demonstrated atypical lymphoid infiltrate composed of medium to large‐sized lymphocytes with irregular nuclear contours, vesicular chromatin, prominent nucleoli, and a moderate amount of cytoplasm (Figure 1A). Flow cytometric analysis showed an abnormal lymphocyte population (Figure 1B) that was unequivocally CD19+ (Figure 1C) and predominantly CD5− (small subset positive) (Figure 1D), suggestive of a B‐cell lymphoma. However, further analysis showed the entire CD19+ population was also brightly positive for CD3, CD7, CD8, and CD2 (Figure 1E–G). This population was clonal based on TRBC1 expression (TRBC1−) (Figure 1H). Immunohistochemistry confirmed the lymphoma was positive for CD19 (Figure 2A), CD3 (Figure 2B), CD8 (Figure 2C), BetaF1, and TIA1, while negative for CD30, CD20 (Figure 2D), and other B‐cell markers including PAX5 (Figure 2E), CD22, CD79a, and OCT2. The possibility of a composite lymphoma or dual cell population was considered; however, flow cytometric analysis revealed a single abnormal cell population that showed co‐expression of CD19, CD3, CD8, and multiple T‐cell markers and was negative for CD20 and surface kappa or lambda light chain (not shown). Tissue immunohistochemical evaluation confirmed the flow cytometry findings. Therefore, the possibility of composite B/T cell lymphoma is excluded. PCR studies were positive for clonal TCR‐beta gene rearrangement and negative for clonal IgH gene rearrangement. A diagnosis of peripheral T‐cell lymphoma, NOS, cytotoxic subtype, with aberrant CD19 expression was established. It is not clear whether this lymphoma represents a transformation of the prior CD30+ CTCL or a separate T‐cell lymphoma as we do not have TCR gene rearrangement data from the prior CD30+ CTCL from 2019 to confirm if they are clonally related. But immunophenotypically, this lymphoma is very different from that of the prior CTCL, which was CD30+, CD4+, CD8−, and CD7−. The lymphoma progressed quickly with extensive involvement of skin, liver, and bone marrow. The patient succumbed to the disease 13 months after this diagnosis despite multiple therapies and hematopoietic stem cell transplant.

**FIGURE 1:**
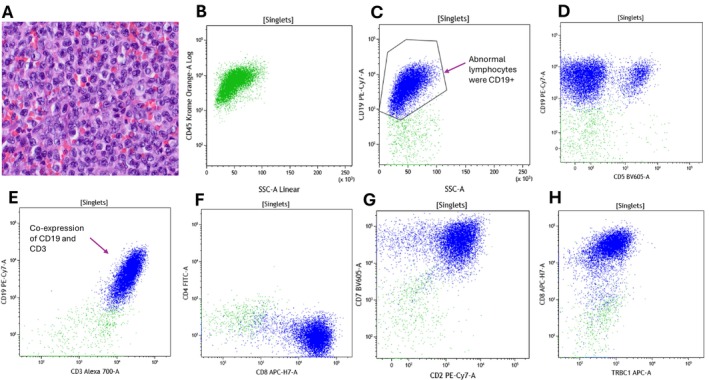
Hematoxylin and eosin stain showing sheets of atypical lymphoid cells (Panel A, 50× objective). Flow cytometric analysis was performed on the cell suspension prepared from the tissue. After gating on viable cells and singlets, a population of CD45+ abnormal lymphoid cells was identified (Panel B, green). The abnormal lymphocytes were unequivocally positive for the B‐cell marker CD19; the mean fluorescence intensity (MFI) value is more than 10 times higher than the negative controls (Panel C, blue). The CD19+ cells are largely negative for CD5 (small subset positive) (Panel D). Further analysis of this population showed co‐expression with T‐cell markers CD3, CD8, CD7, and CD2 (Panel E–G). This population was TRBC1 negative (clonal) (Panel H). Antibodies used in flow cytometry: CD45 KrO (Beckman Coulter, clone J33), CD19 PeCy7 (Beckman Coulter, clone J3‐119), Kappa FITC (Dako), Lambda PE (Dako), CD3 AF700 (Biolegend, clone UCHT1), CD5 BV605 (Biolegend, clone UCHT2), CD4 FITC (BD, clone SK3), CD8 APC‐H7 (BD, clone SK1), CD2 PeCy7 (Beckman Coulter, clone 39C1.5), CD7 BV605 (BD, clone M‐T701), and TRBC1 APC (Biolegend, clone JOVI.1).

**FIGURE 2:**
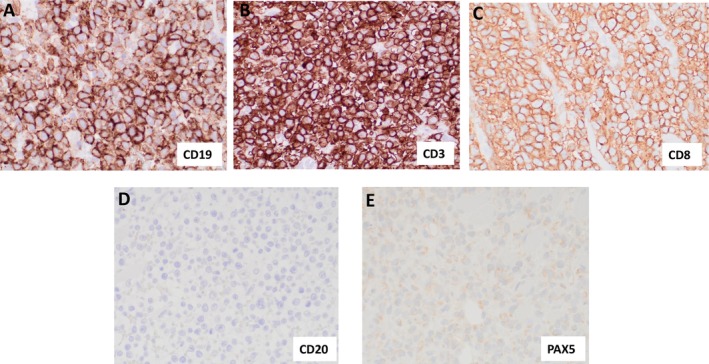
Immunohistochemical staining results. Panel A, immunohistochemistry CD19 showing moderate to strong membranous staining, 50× objective; Panel B, immunohistochemistry CD3 showing strong staining, 50× objective; Panel C, immunohistochemistry CD8 showing positive staining, 50× objective; Panel D, immunohistochemistry CD20, 50× objective; Panel E, immunohistochemistry PAX5, 50× objective.

Expression of B‐cell markers such as CD20 on T‐cell lymphoma has been described [1, 2], but expression of CD19 on T‐cell lymphoma is exceedingly rare [3]. This case illustrates the rare phenomenon of CD19 expression in T‐cell lymphoma, which could pose a significant diagnostic challenge. Comprehensive immunophenotypic and genetic evaluations are critical in reaching the correct diagnosis.

## Author Contributions

B.V., Y.L., and Q.C. were responsible for data collection, interpretation, and creating the manuscript.

## Funding

The authors have nothing to report.

## Ethics Statement

The authors have nothing to report.

## Consent

The authors have nothing to report.

## Conflicts of Interest

The authors declare no conflicts of interest.

## Data Availability

The data that support the findings of this study are available from the corresponding author upon reasonable request.
